# Dispensing by Nurses

**Published:** 1898-11-26

**Authors:** 


### DISPENSING BY NURSES.

At an inquest held at Kingston recently on a man who died at the Tolworth Isolation Hospital from an overdose of opium administered to him while suffering from typhoid fever, the jury expressed their opinion that much greater care should be exercised in dispensing poisonous drugs. In this the coroner quite agreed, but we would go further and express the opinion that much greater care should be exercised in the ordering of drugs when it is understood that they are to be dispensed by nurses and other persons who are not trained pharmacists. From the evidence it appears that the medical officer to the hospital left a written order that the deceased was to have half a drachm of tincture of opium by enema, the order being written on the card. The matron appears to have given this card, together with a two-ounce bottle of tincture of opium, to the nurse, and instructed her to administer the dose. At three a.m. the next morning the nurse informed the matron that the patient was under the influence of the narcotic, and gave her to understand that she had misread the symbol "half a drachm" for half an ounce, and had given the patient that quantity. The patient was then insensible, and he died the same day. According to the evidence of the nurse herself, she misread the dose prescribed on the card, and she added "that the only difference between the symbols denoting a drachm and an ounce was that the one had a little curl at the top!" Of course, we all know this "little curl," and how differently it is written by different prescribers. No doubt the jury were right in saying that more care ought to be exercised in dispensing poisonous drugs, but it is clear that in this case the mode in which the drug was ordered was not free from blame, seeing by whom it had to be dispensed. Why cannot medical men take the trouble to write out in plain English what they want? This matter is becoming a very important one, owing to the multiplication of cottage hospitals and small isolation hospitals, much of the medicine given in which must of necessity be dispensed by more or less unskilled hands. When writing prescriptions which, it may fairly be presumed, are fated to be dispensed by properly-educated pharmaceutical chemists, it may be proper enough to make use of signs and symbols, but we venture to think that it is otherwise when giving orders to nurses. The fact is that the training of some nurses has been carried to such a point that medical men are apt to throw upon nurses at large duties and responsibilities which are quite beyond them as a class. Nothing can be more simple than to write a prescription in such a way that it shall at least be as easy to follow as the recipe for a pudding; and wherever the dispensing of drugs is to be done by unskilled persons they should be ordered in some such simple form, especially when they are of a poisonous nature. As to the use of symbols, with the example of the "British Pharmacopœia" before us, in which every formula is printed in English, and throughout which not a single symbol is used, even though the book is intended for physicians and chemists, it is hard to see any excuse for giving orders to nurses in old-fashioned symbols which, as the unfortunate nurse in this case described, are only distinguished from one another by "a little curl at the top." Even among ourselves it is but a silly form of pedantry which leads us to use symbols which belong neither to science nor to commerce—mere survivals of the dark days of medicine. But to use such signs in giving directions to nurses is but to risk the lives of our patients.

